# Effect of *Coptis chinensis* on Biofilm Formation and Antibiotic Susceptibility in *Mycobacterium abscessus*

**DOI:** 10.1155/2020/9754357

**Published:** 2020-11-10

**Authors:** Cheng-Yin Tseng, Mao-Feng Sun, Tsai-Chung Li, Ching-Ting Lin

**Affiliations:** ^1^Graduate Institute of Chinese Medicine, School of Chinese Medicine, China Medical University, 91 Hsueh-Shih Rd, Taichung City 40402, Taiwan; ^2^Section of Infectious Disease, Hsinchu Mackay Memorial Hospital, 690, Sec. 2, Guang-fu Rd., East Dist, Hsinchu City 30071, Taiwan; ^3^Department of Chinese Medicine, China Medical University Hospital, 2 Yu-de Rd, North Dist., Taichung City 40447, Taiwan; ^4^School of Chinese Medicine, China Medical University, 91 Hsueh-Shih Rd, Taichung City 40402, Taiwan; ^5^Department of Public Health, College of Public Health, China Medical University, 91 Hsueh-Shih Rd, Taichung City 40402, Taiwan; ^6^School of Chinese Medicine, China Medical University, 91 Hsueh-Shih Rd, Taichung City 40402, Taiwan

## Abstract

*Mycobacterium abscessus* infections are notoriously difficult to be treated and newer treatment options are required. *Coptis chinensis* (*C. chinensis*) and its main compound berberine are frequently used to treat bacterial and viral infections. In this study, the susceptibility of *M. abscessus* to *C. chinensis* extract and berberine was assessed by minimal inhibitory concentration (MIC) and minimal bactericidal concentration (MBC) evaluation. The effects of *C. chinensis* and berberine on biofilm formation and antibiotic susceptibility in *M. abscessus* were observed. *C. chinensis* at concentrations of MIC (1.5 mg/mL) and 2 × MIC (3.0 mg/mL) and berberine at ½ × MIC (0.125 mg/mL) demonstrated a strong inhibition of biofilm formation. Concentration of *C. chinensis* at ½ × MIC resulted in a significant reduction in MICs of trimethoprim/sulfamethoxazole (TMP/SXT), clarithromycin (CLA), and linezolid (LZD). Similarly, ½ × MIC berberine had a significant effect on the MIC reductions of nine antibiotics including TMP/SXT, CLA, and LZD. Notably, the resistance level MIC of LZD against *M. abscessus* was reversed to a susceptible level by treatment with either *C. chinensis* or berberine. Therefore, *C. chinensis* and berberine have the potential to produce a synergistic antimycobacterial effect, reduce biofilm formation, and decrease antibacterial resistance to LZD in *M. abscessus*.

## 1. Introduction

Nontuberculous mycobacteria (NTM), also known as environmental mycobacteria, are ubiquitous in a variety of natural habitats (wet soil, marshlands, streams, rivers, and estuaries) and man-made habitats (canals, pastures, and drinking water distribution systems). To date, more than 172 NTM species have been identified [1–3]. Although most species are nonpathogenic, Thomson et al. found pathogenic NTM species in large urban water distribution systems in Australia [4]. Human NTM diseases (e.g., pulmonary diseases, lymphadenitis, skin and soft tissue infections, and disseminated diseases) are believed to result from environmental exposure, trauma, and surgical or cosmetic procedures [5]. In 2019, Hung et al. reported a cluster of patients with postoperative *M. abscessus* endophthalmitis [6]. Wentworth et al. reported a threefold increase in the incidence of cutaneous NTM infections from 1980 to 2009 [7]. A study in Taiwan reported an average of 12.5 cases of NTM skin and soft tissue infection per year, which was an increase from previous years [8]. The prevalence of NTM infections in immunocompromised patients, including AIDS patients and transplant recipients, is growing worldwide [2, 9–11]. The number of NTM disease-related deaths is also rising, with higher mortality rates seen among adults over the age of 55 years and those with chronic obstructive pulmonary disease, bronchiectasis, HIV, interstitial lung diseases, and/or tobacco use [12].

While there is a wide range of treatment strategies available with multidrug antibiotic therapy being the current treatment of choice [13, 14], the results of antibiotic treatment are often discouraging [14]. Patients require a prolonged antibiotic course, which often results in side effects, and/or acquired antibiotic resistance. Suggested treatment is antibiotic therapy lasting 9–18 months, or even up to 24 months [15]. Interestingly, *M. abscessus* infections are widely seen hard to be treated despite the use of antibiotics. In a study of antibiotic therapy for pulmonary *M. abscessus* infections over a median duration of 12 months (range 9–18 months) by Novosad et al., 62% of patients experienced side effects that resulted in the need to adjust the therapeutic regimen [14]. Adjuvant or alternative therapies may be required, and a response to antibiotic side effects is needed. Adding Chinese herbal medicine (CHM) to antibiotic treatments may provide an effective solution for treating antibiotic side effects.

CHM has been used to treat infectious diseases since ancient times in China. Over thousands of years, many mechanisms have been proposed and countless reports of CHM antimicrobial therapy have been published in scientific publications and textbooks, such as the classic Chinese medical monographs “*Treatise on Febrile Diseases (Shāng Hán Lùn)*” and “*Systematic Differentiation of Warm Diseases (Wēn Bìng Tiáo Biàn)*.” In recent decades, a large number of studies have reported on the anti-inflammatory and antimicrobial effects of heat-clearing Chinese herbs (HCCHs) [16–18]. Currently, HCCHs and CHM formulations are frequently used as monotherapy or combined therapy components to treat common infectious diseases such as pulmonary tuberculosis, methicillin-resistant *Staphylococcus aureus* (MRSA) infection, and sepsis [19–21]. CHM formulations with bacteriostatic activity may be useful in treating *M. abscessus* infections.

*Coptis chinensis* (*C. chinensis*; *Huang Lian*) is a traditional CHM frequently used to treat bacterial and viral infections. It has proven efficacy against many microorganisms including *Staphylococcus* spp. [18]. In 2006, Lin et al. found that *Coptidis Rhizoma* (the dried rhizome of *C. chinensis* Franch) provided 100% *in vitro* inhibitory effects on *S. aureus* and *Escherichia coli* [22]. Of note, *C. chinensis* was reported to have an effect comparable to that of the commercial antibiotic vancomycin against coagulase-negative *Staphylococcus* [23]. Recently, *C. chinensis* was found to produce significant antimicrobial activity against a variety of microorganisms including Gram-positive and Gram-negative bacteria [16]. Over 120 chemical components have been isolated from *C. chinensis*, with alkaloids as the main active ingredients and berberine as the most representative component [24]. Berberine's broad-spectrum antibacterial effect is similar to that of *C. chinensis*, with effects manifesting proportional to concentrations. Higher concentrations of berberine are reported to kill bacteria more quickly [25]. In this era of increasing antibiotic resistance, the potential benefits of using *C. chinensis* and berberine in the treatment of *M. abscessus* infections warrant investigation. The aims of this study were to examine *in vitro* the antibacterial effect of *C. chinensis* and berberine (its major constituent) in treating *M. abscessus* infections. The researchers studied the potential of this herb to inhibit biofilm formation and amplify the activity of antibiotics in managing *M. abscessus* infections.

## 2. Materials and Methods

For this study, the *M. abscessus* strain, which was purchased from the College of American Pathologists for qualification of Medical Technologist, from the stock of Medical Laboratory of the Tamshui Branch of Mackay Memorial Hospital in Taiwan, was chosen to represent rapidly growing mycobacterium (RGM). An incubation broth (7H9) and commercially available antimycobacterial susceptibility test kit (Sensititre^TM^ RAPMYCOI, Trek Sensititre, ThermoFisher Scientific) were used to test the susceptibility of the RGM to a panel of 15 antibiotics which is recommended by the Clinical and Laboratory Standards Institute (CLSI) [26]. For the purpose of comparing the actual inhibitory activities of pretreatment (antibiotic alone) and combination treatment (antibiotic plus *C. chinensis* or berberine), the Sensititre^TM^ RAPMYCOI was further used to test the synergistic antibacterial effects of *C. chinensis* and berberine. Based on our review of Chinese Medicine books, research reports, and discussions with traditional Chinese medicine physicians, we selected the CHM *C. chinensis* for this study, an herb containing 6.19% berberine. In order to overcome the possible issues, such as sample preparation or different batch of raw material, and to make the results more reliable, a pharmacy type of concentrated *C. chinensis* paste which was approved for clinical use by the Ministry of Health and Welfare, Taiwan (provided by Kaiser Pharmaceutical Co., Ltd; product: 8010) was used. The herbal materials provided by Kaiser Pharmaceutical conformed to the standards of Taiwan Herbal Pharmacopeia 2. The procedures complied with the drugs registration license approved by the Taiwan Food and Drug Administration, in which the extraction of the raw material is performed exclusively using water. Additionally, the concentrations of commercially available berberine and berberine-HCl obtained from the products of *C. chinensis* were tested by Kaiser Pharmaceutical by HPLC following the protocol listed in Taiwan Herbal Pharmacopeia 2. The antimicrobial activities of commercially available berberine and berberine-HCl were identical as examined with its antibacterial activities against *M. abscessus.* Thus, the erstwhile described berberine and berberine-HCl were purchased to enable a detailed analysis of its potential to enhance antibacterial effects (ISO 9001: 2015 certification; http://www.chemfaces.com).

### 2.1. *M. abscessus* Susceptibility to *C. chinensis* and Berberine

*C. chinensis* was dissolved in 7H9 broth and then serially diluted twofold from 12.0 mg/mL for testing concentration from 12.0 mg/mL to 0.375 mg/mL. The antibacterial effects of berberine against *M. abscessus* were determined in the same manner, from 2.0 mg/mL to 0.0625 mg/mL. The antibacterial activity of *C. chinensis* and berberine was evaluated by counting the colony forming unit (CFU) of *M. abscessus* found after their administration. As the protocol of TREK Diagnostic Systems, *M. abscessus* suspension (optical OD_600_ ∼0.2) was then added to the indicated concentrations of *C. chinensis* and berberine-HCl to observe the bacterial growth at 30°C for 5 days [27]. Relative percentage of survival rate was expressed as CFU of viable bacteria present after treatment with the indicated concentrations of *C. chinensis* or berberine-HCl relative to the number of viable bacteria present at untreatment and then multiplied by 100.

### 2.2. Effects of *C. chinensis* and Berberine on the Biofilm Formation of *M. abscessus*

To evaluate the prevention effect before biofilm development, serial indicated concentrations of *C. chinensis* and berberine-HCl were individually added to the glass test tubes in the same time of preparation of *M. abscessus* solution to calculate optical density differences. Optical densities before and after the addition of *C. chinensis* and berberine-HCl were measured and compared.

Biofilm formation was assessed by the ability of cells to adhere to the glass tubes, with some modification of the reported protocol [28]. For testing, the concentration of *C. chinensis* ranging between 3 and 0.375 mg/mL, and the concentration of berberine ranged between 0.5 and 0.0625 mg/mL. The glass test tube contained a 1 : 10 diluted aliquot of *M. abscessus* overnight culture, which was incubated in 7H9 broth with different concentrations of *C. chinensis* and berberine-HCl at 30°C for 14 days. After 14 days, nonadherent *M. abscessus* was triple washed with 3 mL PBS, and the adherent *M. abscessus* was stained with 3 mL of 0.1% safranin solution at room temperature for 30 min. The glass tube was rinsed twice with deionized water to remove excess stain. Finally, the safranin stained biofilm was solubilized in 1 mL of 33% acetic acid, and then 100 *μ*L of the solution was removed to 96-well PVC plates (TPP 96 flat) for measuring the absorbance at *λ* = 492 nm.

### 2.3. Effects of *C. chinensis* and Berberine on Minimal Inhibitory Concentrations (MICs) of Antibiotics against *M. abscessus*

*Mycobacterium abscessus* was treated with 15 antibiotics, without or with *C. chinensis* and berberine-HCl. MICs in this study were determined using a commercially available antimycobacterial susceptibility test kit (Sensititre^TM^ RAPMYCOI, Trek Sensititre, ThermoFisher Scientific) according to the manufacturer's instructions. Briefly, 50 *μ*L of the *M. abscessus* suspension, grown in 7H9 broth at 30°C for 5 days and adjusted to optical OD_600_ ∼ 0.2, was added to 11 ml 7H9 broth alone or containing the indicated concentrations of *C. chinensis* (3 mg/mL to 0.375 mg/mL) or berberine-HCl (0.5 mg/mL to 0.0625 mg/mL) and mixed well. Then, 100 *μ*L of the mixture was added per well of the kit's Sensititre^TM^ Myco RAPMYCOI AST Plate, with evaluated concentrations of 15 antibiotics as (*μ*g/ml) trimethoprim/sulfamethoxazole, 0.25/4.75–8/152; linezolid, 1–32; ciprofloxacin, 0.12–4; imipenem, 2–64; moxifloxacin, 0.25–8; cefepime, 1–32; cefoxitin, 4–128; amoxicillin/clavulanic acid, 2/1–64/32; amikacin, 1–64; ceftriaxone, 4–64; doxycycline, 0.12–16; minocycline, 1–8; tigecycline, 0.015–4; tobramycin, 1–16; and clarithromycin, 0.06–16, for incubating at 30°C for 5 days. Finally, we observed the effect of *C. chinensis* and berberine-HCl on the MICs of 15 antibiotics against *M. abscessus*. Both the MICs of the antibiotics alone and the MICs of the antibiotics plus *C. chinensis* and berberine-HCl were measured at least three times.

### 2.4. Statistical Analysis

The CFU numbers of *M. abscessus* were individually calculated after the indicated concentrations of *C. chinensis* and berberine-HCl were added. The results were expressed as relative percentages ± standard deviations (SD). The effects on biofilm formation after *C. chinensis* and berberine-HCl treatment were also analyzed. The results were expressed as mean scores ± SD. The Mann–Whitney Rank Sum Test was conducted to examine any differences between control and experimental groups with indicated concentrations. The level of statistical significance was set at *p* < 0.05.

## 3. Results

### 3.1. Susceptibility of *M. abscessus* to *C. chinensis* and Berberine

**Figure 1:**
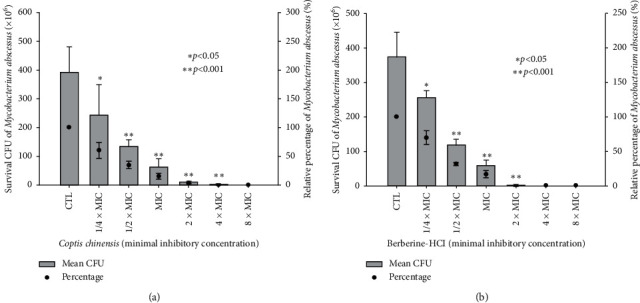
Susceptibility of *M. abscessus* to *C. chinensis* or berberine-HCl. The CFU formed of *M. abscessus* in 7H9 medium alone (the control group) and after treatment with the indicated concentrations of *C. chinensis* or berberine-HCl were counted by plating. Survival CFU and the relative percentage were expressed. As determined by the Mann–Whitney Rank Sum Test, statistically significant reductions (asterisk) in relative percentage of survival CFU of *M. abscessus* were observed for 1/4 × MIC, 1/2 × MIC, MIC, 2 × MIC, and 4 × MIC following the addition of *Coptis chinensis* (a) and 1/4 × MIC, 1/2 × MIC, MIC, and 2 × MIC following the addition of berberine-HCl (b). CFU: colony forming unit. CTL: control. MIC: minimal inhibitory cncentration. Error bars indicate standard deviations. ^*∗*^*p* < 0.05 and ^*∗∗*^*p* < 0.001.

For evaluation of the effects of *C. chinensis* on the growth of *M. abscessus*, the number of *M. abscessus* CFU formed in 7H9 medium with different concentrations of *C. chinensis* was calculated and compared to the number of *M. abscessus* colonies formed in 7H9 medium alone (the control group). The relative survival percentages of *M. abscessus* were 59.95 ± 13.70%, 34.56 ± 6.58%, 15.20 ± 4.74%, and 2.55 ± 0.27% at concentrations of 0.375 mg/mL, 0.75 mg/mL, 1.5 mg/mL, and 3.0 mg/mL, respectively (Figure 1(a)). Therefore, the MIC value of *C. chinensis* was around 1.5 mg/mL for *M. abscessus*. Additionally, we observed that the growth of *M. abscessus* approached zero at *C. chinensis* concentration of 6.0 mg/mL (0.31 ± 0.34%). This implies that minimal bactericidal concentration (MBC) was 4 times higher than MIC of *C. chinensis*. The reductions in relative percentage of survival CFU of *M. abscessus* were statistically significant for 1/4 × MIC, 1/2 × MIC, MIC, 2 × MIC, and 4 × MIC following the addition of *C. chinensis*. Our findings revealed that *C. chinensis* has a conspicuous bacteriostatic effect on *M. abscessus*.

Berberine is the main active component of *C. chinensis*. To further observe the effects of berberine on *M. abscessus* growth, the CFU numbers of *M. abscessus* with different concentrations of berberine-HCl were calculated and compared to the numbers of *M. abscessus* grown in 7H9 medium alone. The relative survival percentages of *M. abscessus* were 69.45 ± 9.68%, 31.41 ± 2.50%, 16.27 ± 4.72%, and 0.57 ± 0.62% at berberine-HCl concentrations of 0.0625 mg/mL, 0.125 mg/mL, 0.25 mg/mL, and 0.5 mg/mL, respectively (Figure 1(b)). The MIC value of berberine-HCl was around 0.25 mg/mL for *M. abscessus*. Likewise, nearly no *M. abscessus* growth was observed with berberine-HCl concentrations in 0.5 mg/mL and in excess of 1.0 mg/mL. The MBC of berberine-HCl was 2–4 times higher than MIC. As determined by the Mann–Whitney Rank Sum Test, the differences in relative percentage of survival CFU of *M. abscessus* between pretreatment and the addition of 1/4 × MIC, 1/2 × MIC, MIC, and 2 × MIC berberine were statistically significant. Our observations indicate that berberine-HCl also showed an obvious bacteriostatic effect against *M. abscessus* growth.

### 3.2. Effects of *C. chinensis* and Berberine on the Biofilm Formation of *M. abscessus*

**Figure 2:**
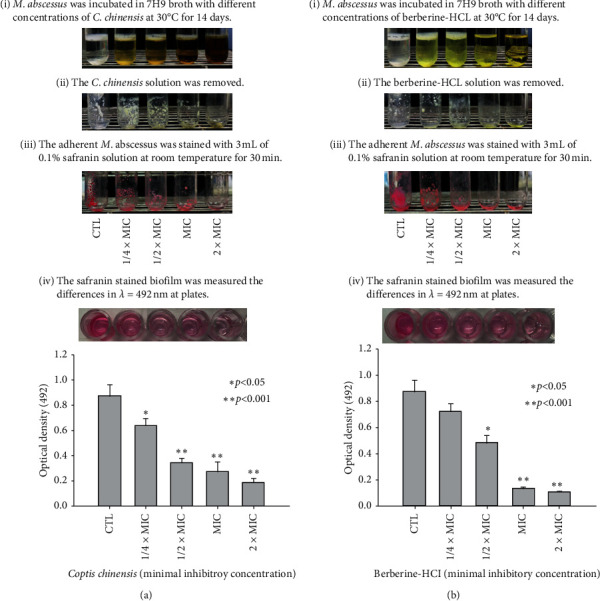
Comparative effects of indicated concentration of *C. chinensis* and berberine-HCl on the biofilm formation of *M. abscessus*. *M. abscessus* was grown in 7H9 broth (control) and under the concentrations of ¼ × MIC, ½ × MIC, MIC, and 2 × MIC of *C. chinensis* (a) and berberine-HCl (b) at 30°C for 14 days. *M. abscessus* solution was incubated at 30°C for 14 days (i). After 14 days, nonadherent *M. abscessus* was removed (ii). The adherent *M. abscessus* was stained with 3 mL of 0.1% safranin solution at room temperature for 30 min (iii). The safranin stained biofilm was solubilized in acetic acid and then was removed to 96-well PVC plates for measuring the differences in optical density 492 (*λ* = 492 nm) (iv). The photo represented biofilm solutions of control and indicated concentrations of *C. chinensis* and berberine-HCl. Mean values of three independent experiments and standard deviations were shown. The Mann–Whitney Rank Sum Test was used to calculate statistical significance. An asterisk indicates a significant difference between the control group and indicated concentration groups. Statistically significant inhibition on the biofilm formation of *M. abscessus* was observed for 1/4 × MIC, 1/2 × MIC, MIC, and 2 × MIC concentrations of *C. chinensis* and 1/2 × MIC, MIC, and 2 × MIC concentrations of berberine-HCl. CTL denotes control. MIC denotes minimal inhibitory concentration.

The biofilm formation of *M. abscessus* on the glass tubes was reviewed and compared. The biofilm of *M. abscessus* declined after incubation with *C. chinensis* and berberine-HCl for 14 days. The effects manifested were proportional to the added concentrations. Higher concentrations of *C. chinensis* and berberine-HCl exhibited poor abilities of adherence. To analyze whether *C. chinensis* and berberine-HCl affect biofilm formation of *M. abscessus*, the biofilm was determined at OD_492_ by staining with safranin. As shown in Figure 2(a), the differences of optical density 492 were (mean ± standard deviation) 0.872 ± 0.0903, 0.641 ± 0.0530, 0.344 ± 0.0348, 0.275 ± 0.0767, and 0.190 ± 0.0312 at control and concentrations of ¼ × MIC, ½ × MIC, MIC, and 2 × MIC of *C. chinensis*, respectively. We found that the concentrations of *C. chinensis* at 1/4 × MIC, 1/2 × MIC, MIC, and 2 × MIC significantly decreased *M. abscessus* biofilm formation. These results indicate that the biofilm formation of *M. abscessus* could be inhibited by the concentration of *C. chinensis* in excess of 1/4 × MIC. When indicated MICs of berberine-HCl were added (Figure 2(b)), the differences of optical density 492 were (mean ± standard deviation) 0.872 ± 0.0903, 0.723 ± 0.0562, 0.483 ± 0.0580, 0.137 ± 0.00832, and 0.110 ± 0.00362 at control and concentrations of ¼ × MIC, ½ × MIC, MIC, and 2 × MIC, respectively. We observed that biofilm formation of *M. abscessus* slightly decreased at ¼ × MIC and considerably decreased at ½ × MIC, MIC, and 2 × MIC. These findings reveal that biofilm formation of *M. abscessus* could be consistently inhibited using berberine-HCl concentrations in excess of ½ × MIC.

### 3.3. Effects of *C. chinensis* on the MICs of Antibiotics against *M. abscessus*

According to the reference MICs of antibiotics for *M. abscessus*, this study strain of *M. abscessus* was primarily resistant to most studied antibiotics except clarithromycin, tigecycline, and amikacin [26, 29]. To observe whether *C. chinensis* would affect the MICs of antibiotics against *M. abscessus*, combinations of antibiotics and *C. chinensis* were tested. The MICs of antibiotics alone and antibiotics combined with *C. chinensis* were then compared. The addition of *C. chinensis* in concentrations of ¼ × MIC, ½ × MIC, MIC, and 2 × MIC were tested to measure the effects on MICs of antibiotics. As shown in Table 1, the addition of ¼ × MIC *C. chinensis* provided a reduction of only MIC in linezolid. *C. chinensis* concentrations of ½ × MIC and MIC resulted in significant synergistic effects measured for trimethoprim/sulfamethoxazole, clarithromycin, and linezolid. Importantly, the synergistic effect of *C. chinensis* on these three antibiotics is highly related to the concentration of *C. chinensis*. Furthermore, the concentration of *C. chinensis* at MIC could also reduce the MICs of imipenem and cefoxitin. We observed that the growth of *M. abscessus* was entirely inhibited by combining *C. chinensis* at the concentration of 2 × MIC with each of the 15 antibiotics tested in this study. Therefore, *C. chinensis* could significantly amplify the antibacterial effects of trimethoprim/sulfamethoxazole, clarithromycin, and linezolid against *M. abscessus*. This effect was proportional to the *C. chinensis* concentration.

### 3.4. Effect of Berberine on the MICs of Antibiotics against *M. abscessus*

The impact of berberine-HCl on the MICs of antibiotics against *M. abscessus* was also assessed. Berberine-HCl concentrations of ¼ × MIC, ½ × MIC, MIC, and 2 × MIC were added to commercial 96-well plates to measure the MICs of 15 antibiotics. Notably, the growth of *M. abscessus* was entirely inhibited with the addition of berberine-HCl at concentrations of MIC and 2 × MIC in the 15-antibiotic well plates. As shown in Table 2, berberine-HCl concentration at ¼ × MIC only reduced the MIC of trimethoprim/sulfamethoxazole. Interestingly, we observed reductions of MICs in 9 of the 15 (60%) antibiotics that were tested (trimethoprim/sulfamethoxazole, clarithromycin, linezolid, amikacin, tigecycline, imipenem, cefepime, cefoxitin, and ciprofloxacin) when combined with berberine-HCl at a concentration of ½ × MIC. Similar to the effects of *C. chinensis*, the synergistic effects of berberine-HCl were more significant with trimethoprim/sulfamethoxazole, clarithromycin, and linezolid. These observations indicate that berberine-HCl can enhance the antimycobacterial activity of most antibiotics against *M. abscessus*.

## 4. Discussion

The development of novel antimycobacterial drugs is ongoing. The antimicrobial, anti-inflammatory, and synergistic antibacterial effects of CHMs have been demonstrated in several studies [16, 20, 21, 30, 31]. Single extracts or formulations of CHM alone, or in combination with antibiotics, may be used to treat common bacterial infections such as MRSA, *Ureaplasma urealyticum*, and genital *Chlamydia trachomatis* infections [20, 32]. In 2007, Liu et al. reported that 26 of 58 (44.8%) CHMs used in Taiwan exhibited antibacterial activity against antibiotic-resistant *Pseudomonas aeruginosa,* and one of the studied herbal medicines (*Ramulus Cinnamomi*) could yield a synergistic effect with antibiotics [30]. In the current study, we demonstrated that *C. chinensis* and its major constituent, berberine, can inhibit the growth of *M. abscessus*. The MICs of *C. chinensis* and berberine-HCl were 1.5 mg/mL and 0.25 mg/mL, respectively.

The formation of biofilm is a key mechanism in the pathogenicity and resistance of human NTM infections. Bacteria within a biofilm exhibit higher virulence to human and resistance to antimicrobials and disinfectants. Complete differentiation, achievement of maximal replication, horizontal gene exchanges and mutations, and more adaption to environment were reported [2, 33]. In addition, it influences the effectiveness of endoscope and invasive device disinfection by automated processors in hospitals [34, 35]. Biofilm formation is usually an important factor in antimicrobial resistance and can lead to treatment failure. In 2011, Lin et al. reported that an active ingredient in commonly used Chinese herbal plants had inhibitory effects on *S. aureus* biofilm formation [36]. The capability of *C. chinensis* and berberine to reduce biofilm formation by *M. abscessus* was proven in this study. Biofilm is a complex multicellular structure that forms on bacterial surfaces. The complexity of mycobacterial biofilm, the synthesis of an extracellular matrix, usually involves various molecules including glycopeptidolipids, DNA such as groEL1 gene, and mycolic acids. The process of biofilm development is also influenced and regulated by nutrients, ions, and carbon sources [37]. Some proteins, such as adhesins, are believed to potentiate the aggregation of mycobacteria and their ability to bind to other cells [38]. Mycolic acids were reported to contribute to the resistance of mycobacteria to antibiotics and disinfectants [39]. The detailed mechanisms of reducing biofilm formation in *M. abscessus* for *C. chinensis* and berberine need further investigation.

Antimicrobial therapies for *M. abscessus* infections have been investigated for decades. However, treatment regimens vary in duration, tolerability, and success. Clarithromycin, amikacin, and cefoxitin are the most commonly reported antimycobacterial choices for the treatment of *M. abscessus* infections [14]. Macrolide-based regimens in combination with intravenously administered antimicrobials are the currently recommended therapy [40]. However, poor responsiveness to antimicrobial chemotherapy, including macrolide-based regimens for *M. abscessus* infections, remains a challenging problem. Furthermore, side effects such as gastrointestinal upset, skin changes, and renal insufficiency are common and frequently require changing or discontinuing antibiotic therapy [14].

Previous literature has reported that combining antibiotics with CHM can produce better curative effects when treating pulmonary tuberculosis [19]. Treating intractable mycobacteriosis with antibiotics and CHMs that exhibit immunoadjunctive effects has been reported with favorable outcomes [41]. Likewise, the synergistic effects of the herbal plant extract *Thymbra spicata* L. and antibiotics against common, multidrug-resistant *S. aureus,* and *Klebsiella pneumoniae* strains were reported in 2016 [31]. Similar antibacterial effects of antibiotics in combination with Chinese herbal remedies for *M. abscessus* infections can be expected, but currently there are no such reports in the literature.

*C. chinensis* and berberine-HCl were proven to inhibit *M. abscessus* growth in a dose-dependent manner. Additionally, both *C. chinensis* and berberine-HCl significantly strengthened the antimicrobial activities of trimethoprim/sulfamethoxazole, clarithromycin, and linezolid against *M. abscessus*. According to measurements taken in this study, the MIC of linezolid was 32 *μ*g/mL, defined as resistant to antimicrobial susceptibility for *M. abscessus*. The additions of both of these CHMs reduced its MIC to 4 *μ*g/mL, which is categorized as a susceptible level of linezolid against *M. abscessus*. Both parenteral and oral linezolid preparations are available for clinical use. The clinical implications of the combination of linezolid and *C. chinensis* or berberine may be prospective and therefore are recommended for long-term treatment of *M. abscessus* infections.

Although we found that berberine demonstrated an antimycobacterial effect on *M. abscessus*, the antibacterial mechanism of berberine on *M. abscessus* growth remains unknown, although previous studies have provided some useful information. Peng et al. reported that berberine could damage the cell membrane and walls of *Streptococcus agalactiae*, thereby increasing membrane permeability, and inhibit protein and DNA synthesis in cells [25]. Amin et al. also reported that berberine could inhibit RNA and protein synthesis in *E. coli* and exhibits antimicrobial effects [42]. The authors suggest that both Gram-positive and Gram-negative bacteria are susceptible to berberine. Therefore, whether berberine could inhibit the growth of *M. abscessus* due to membrane integrity disruption or nucleotide acid or protein synthesis inhibition needs to be investigated further.

The antimicrobial activity of trimethoprim/sulfamethoxazole is due to its interference with folate synthesis. The antimicrobial activity of clarithromycin and linezolid is due to their inhibition of protein synthesis by binding to 50S subunits of bacteria [43, 44]. Therefore, the synergistic effects of both *C. chinensis* and berberine-HCl in combination with these three antibiotics may be because of their interference with protein and RNA synthesis, as well as the cellular membrane. In addition, combining berberine-HCl with antibiotics could amplify the antimicrobial activities of amikacin and tigecycline through the mechanisms mentioned above. In contrast, the MIC reductions of amikacin, tigecycline, ciprofloxacin, and cefepime, measured when they were combined with 0.125 mg/mL (½ × MIC) berberine-HCl were not observed with *C. chinensis*. When compared with berberine, the attenuation of antimycobacterial activity in antibiotics is hypothesized to be antagonistic to other *C. chinensis* components. Moreover, we found that *C. chinensis* increases the MIC of tigecycline, which acts as a protein synthesis inhibitor by binding with 30S ribosomal subunits of bacteria [45]. The relationship between *C. chinensis* and the 30S ribosomal protein subunits of bacteria needs to be investigated further. Integrating the pleomorphic effects of *C. chinensis* and its active component berberine may contribute to our knowledge of pathways and enhance the susceptibility of *M. abscessus* to multiple antibiotics. Therefore, this herb and its main component could be used and tested as an adjunct therapy for *M. abscessus* infections.

## 5. Conclusions

The results of this study demonstrate that combination treatment can enhance the effects of certain antibiotics against *M. abscessus*. Chinese and Western combination treatments deserve more attention because they can be convenient and safe. The combinations of *C. chinensis* or berberine with the antibiotics identified in this study may represent a novel strategy for the treatment of patients with *M. abscessus* infections.

## Figures and Tables

**Table 1:** Minimal inhibitory concentrations of antibiotics alone and in combination with *Coptis chinensis* in different concentrations to treat *Mycobacterium abscessus*.

Antibiotic (target)	Minimal inhibitory concentration (*μ*g/mL)				
	Antibiotic alone	*Coptis chinensis*			
		¼ × MIC	½ × MIC	MIC^a^	2 × MIC
Folate					
TMP/SXT	>8/152	>8/152	8/152	4/76	NG
Protein (50S)					
CLA	0.5	0.5	0.25	0.12	NG
LZD	32	16	4	4	NG
Protein (30S)					
AMI	16	16	16	16	NG
TOB	16	16	16	16	NG
DOX	>16	>16	>16	>16	NG
MIN	>8	>8	>8	>8	NG
TGC	0.5–1	1	2	2	NG
Cell wall					
IMI	64	64	64	32	NG
FOX	64	64	64	32–64^b^	NG
FEP	>32	>32	>32	>32	NG
AXO	>64	>64	>64	>64	NG
AUG	>64/32	>64/32	>64/32	>64/32	NG
Nucleic acid					
CIP	>4	>4	>4	>4	NG
MXF	>8	>8	>8	>8	NG

^a^MIC = 1.5 mg/mL. ^b^One data point was measured at 64 *μ*g/ml. TMP/SXT, Trimethoprim/sulfamethoxazole; CLA, clarithromycin; LZD, linezolid; IMI, imipenem; FOX, cefoxitin; CIP, ciprofloxacin; MXF, moxifloxacin; DOX, doxycycline; MIN, minocycline; AMI, amikacin; TOB, tobramycin; AUG, amoxicillin/clavulanic acid; AXO, ceftriaxone; FEP, cefepime; TGC, tigecycline; NG, no growth.

**Table 2:** Minimal inhibitory concentrations of antibiotics alone and in combination with berberine-HCl at different concentrations to treat *Mycobacterium abscessus*.

Antibiotic (target)	Minimal inhibitory concentration (*μ*g/mL)				
	Antibiotic alone	Berberine-HCl (mg/mL)			
		¼ × MIC	½ × MIC	MIC^a^	2 × MIC
Folate					
TMP/SXT	>8/152	8/152	4/76	NG	NG
Protein (50S)					
CLA	0.5	0.5	0.12	NG	NG
LZD	32	32	4	NG	NG
Protein (30S)					
AMI	16	16	8	NG	NG
TOB	16	16	16	NG	NG
DOX	>16	>16	>16	NG	NG
MIN	>8	>8	>8	NG	NG
TGC	0.5–1	0.5–1	0.25–0.5^b^	NG	NG
Cell wall					
IMI	64	64	32	NG	NG
FOX	64	64	32	NG	NG
FEP	>32	>32	32	NG	NG
AXO	>64	>64	>64	NG	NG
AUG	>64/32	>64/32	>64/32	NG	NG
Nucleic acid					
CIP	>4	>4	4	NG	NG
MXF	>8	>8	>8	NG	NG

^a^MIC = 0.25 mg/mL. ^b^Twofold reduction of MIC for combination with berberine-HCl 0.125 mg/mL. TMP/SXT, trimethoprim/sulfamethoxazole; CLA, clarithromycin; LZD, linezolid; IMI, imipenem; FOX, cefoxitin; CIP, ciprofloxacin; MXF, moxifloxacin; DOX, doxycycline; MIN, minocycline; AMI, amikacin; TOB, tobramycin; AUG, amoxicillin/clavulanic acid; AXO, ceftriaxone; FEP, cefepime; TGC, tigecycline; NG, no growth.

## Data Availability

The data used and/or analyzed during the current study are available from the authors upon request.
